# Mirror Exposure Training for Adolescents With Anorexia Nervosa (MIRADAN): Cognitive Mechanisms of Body Disturbance – A Study Protocol

**DOI:** 10.32872/cpe.11277

**Published:** 2023-12-22

**Authors:** Maarit Pelzer, Jessica Werthmann, Christian Fleischhaker, Jennifer Svaldi, Brunna Tuschen-Caffier

**Affiliations:** 1 Department of Psychology, Clinical Psychology and Psychotherapy, University of Freiburg, Freiburg, Germany; 2 Department of Child and Adolescent Psychiatry, Psychotherapy and Psychosomatics, Freiburg University Hospital, Freiburg, Germany; 3 Faculty of Science, Clinical Psychology and Psychotherapy, Eberhard Karls University Tübingen, Tübingen, Germany; Philipps-University of Marburg, Marburg, Germany

**Keywords:** anorexia nervosa, body dissatisfaction, modifying attentional processes, mirror exposure, adolescence

## Abstract

**Background:**

Anorexia Nervosa (AN) is a severe mental illness, which typically develops in adolescence and, if left untreated, often becomes chronic. Body dissatisfaction is a core characteristic of AN. Mirror exposure (ME) is an effective therapeutic technique to tackle body dissatisfaction in adult patients with eating disorders, but there is limited evidence for the effects of ME in adolescence. One potential mechanism underlying effects of ME on body dissatisfaction is change in body-related attention bias. However, this mechanism remains to be empirically tested. Accordingly, the aim of the current study is twofold: primarily, we aim to test if ME can reduce body dissatisfaction and associated symptoms in adolescent patients with AN. Additionally, we aim to investigate whether change in biased body-related attention due to ME is a possible mechanism of action.

**Method:**

Adolescent patients with AN are randomized to either 12 sessions of ME (3 ME-sessions/week) or wait-list within four weeks. Main outcomes include body dissatisfaction and associated symptoms of AN. Moreover, body-related attention bias is assessed at baseline and post-treatment by means of eye-tracking with two paradigms. Further, process variables are collected weekly. In addition, 12 weeks after end of the study, the acceptability of the ME is assessed.

**Discussion:**

The main aim of the study is to evaluate high-frequency and high-intense ME for treating body dissatisfaction in adolescents with AN. In addition, we would like to clarify whether change in attentional bias for body stimuli is a mechanism underlying change in body dissatisfaction due to ME.

## Highlights

- Body dissatisfaction is a major risk factor for the development, maintenance, and relapse of AN.
- In this study we aim to test if mirror exposure reduces body dissatisfaction in young people with AN.
- A secondary aim is to test if change in body-related attention bias is a mechanism of change in body dissatisfaction.

## Background

Eating disorders are highly prevalent among young women. For example, an 8-year longitudinal study found that 12% of female adolescents experienced some form of threshold or subthreshold eating disorder by age of 20 (Stice et al., 2009). Among people with a lifetime diagnosis of AN with early onsets (< 25 years), about 40% were diagnosed within the age range of 15 to 18 years, making this a prominent age to receive an AN diagnosis (Grilo & Udo, 2021).

AN has the lowest one-year remission rate (Stice et al., 2013) and current treatments of AN in adolescents show only moderate success, leaving room for further improvements (e.g. for an overview of the treatment of AN, Brockmeyer et al., 2018; Jansingh et al., 2020). This is particularly concerning because there was a highly significant increase of 40% in admission rates in the female children’s and the adolescents’ of typical and atypical AN between the pre- and peri-COVID-19 periods in 2019 and 2021, respectively, in Germany (Herpertz-Dahlmann et al., 2022). This illustrates how important it is to improve treatment options for adolescents with AN.

A disturbed body image is a core characteristic of AN. Body image disturbance is characterized by dysfunctional attitudes and emotions toward one's body, such as body dissatisfaction or fear of weight gain (Forrest et al., 2018; Mitchison et al., 2018). Research findings show that overaluation of shape and weight and the corresponding body dissatisfaction is a key risk factor for the development, maintenance, and relapse of AN (Glashouwer et al., 2019; Jacobi et al., 2004). Therefore, decreasing body dissatisfaction in AN is an important treatment target (DuBois et al., 2017).

Several meta-analytic reviews indicated that repeated confrontation with one's own body seems to have a positive influence on body image (Alleva et al., 2015) and that body exposure is an effective intervention for body disturbance in eating disorders (Griffen et al., 2018; Hartmann et al., 2021).

However, while in general ME seems to treat body image disturbances well in people with clinical and subclinical groups, research on the effectiveness of ME in patients with AN is still limited. One uncontrolled study showed that eight weeks of body image therapy with ME exercises in a group format (*n* = 9) compared to body image therapy without ME exercises (*n* = 6) significantly reduced body dissatisfaction, body anxiety and avoidance behaviors (Key et al., 2002). However, the reliability of these results is severely limited due to the particularly low power of the study (Nestoriuc et al., 2012). A larger uncontrolled study of Morgan et al. (2014) (*n* = 55) with exposure-based body image therapy (which included ME in seven out of ten group sessions) yielded significantly lower levels of body-related anxiety and worry, dysfunctional body and eating behaviors compared to baseline. It should be noted here that the two uncontrolled therapy studies by Key et al. (2002) and Morgan et al. (2014) examined patients with AN who were in partial remission with an almost healthy weight (BMI inclusion criterion ≥ 20.5 in Key et al., 2002; BMI inclusion criterion ≥ 17.5 in Morgan et al., 2014). Additionally, a case study showed that patients with AN (*n* = 3) in partial remission benefited from intensive Acceptance and Commitment (ACT) therapy with ME exercises (up to three sessions of 17 therapy sessions in total) in terms of their general pathology, eating symptomatology and body acceptance (Berman et al., 2009).

Additional evidence comes from studies investigating the therapeutic effects of ME in a mixed group of women with AN, Bulimia Nervosa and Eating Disorder Not Otherwise Specified: Results from these studies indicate that negative body-related thoughts and emotions decreased and overall body dissatisfaction and body-related avoidance behavior were reduced by confrontation-based body image therapies in women with eating disorders compared to the control group without therapy (Bhatnagar et al., 2013; Vocks et al., 2008). However, in these studies, ME exercises were part of a broader body image therapy (Bhatnagar et al., 2013: one out of five sessions with predominantly imaginary body exposure exercises; Vocks et al., 2008: three out of ten group therapy sessions), which makes it difficult to attribute effects to ME, specifically.

A functional magnetic resonance imaging (fMRI) study (Vocks et al., 2010) found no effects of body exposure (including ME-sessions) in self-reported measures of AN patients, but reported an increase in the activity of the extrastriate body area from pre- to post-treatment. The authors interpreted this finding as reduction of avoidant body-related processing in response to body image therapy, which may be one working mechanism of ME.

Recently a randomized control trial (RCT) was conducted in which young girls aged 11-17 years with a diagnosis of AN (*n* = 15) received body image therapy, including six ME sessions (out of a total of 14 sessions) as add-on to their inpatient eating disorder therapy; (Biney et al., 2021). Compared to a group without additional body image therapy (*n* = 16, treatment as usual (TAU)), the experimental group showed significantly greater improvements in weight concerns, body-related avoidance behavior and fears of gaining weight. Again, all patients had reached their individual minimum healthy weight prior to body image therapy, including ME sessions. Moreover, the effects, especially for body-related avoidance behavior, observed in this study cannot be attributed to ME specifically, because other body image exercises were also included in the body image therapy received by the experimental group.

To summarize, there are first indications for the effectiveness of ME for patients with AN. However, sample sizes of previous studies were considerably low (ranging between 9 and 15 participants per group) thereby limiting the power of observed effects. Moreover, because ME was mostly applied as a component of a comprehensive “body-related” treatment, there is a lack of reliable randomized controlled data on the specific effect of ME in AN, especially for adolescent patients.

Even though evidence is accumulating the ME may be effective to target body dissatisfaction in AN, it is still unclear why ME may work. Empirical evidence suggests that one potential mechanism underlying body dissatisfaction is an aberrant attention bias to negatively-valenced body parts (for an overview see Jiang & Vartanian, 2018; Kerr-Gaffney et al., 2019; Rodgers & DuBois, 2016) and ME may specifically target this by changing attention processing of one own’s body during repeated confrontations with the own body in the mirror. Accordingly, reducing body-related attention bias may be a working mechanism of ME.

However, experimental evidence for the causal relation of biased attention and body dissatisfaction as potential underlying mechanism of ME remains sparse and contradictory so far (Glashouwer et al., 2016; Krohmer et al., 2022a; Naumann et al., 2022). Initial evidence comes from a study by Smeets et al. (2011) demonstrating that directing attention towards subjectively positive body parts led to a reduction in body dissatisfaction in people with high body dissatisfaction. Similarly, Krohmer et al. (2022a) found that ME improved on body-related attention bias in in the female patients with Binge Eating Disorder compared to the waiting control group. In addition, change in attention bias correlated significantly with change in weight concerns. In a study by Glashouwer et al. (2016), five weeks of ME therapy (one session/week) in which women with high body dissatisfaction were instructed to focus on their subjectively attractive body parts also led to a reduction in self-reported body dissatisfaction. However, in this study, the instruction to direct attention towards subjective attractive body parts did not produce any changes in body-related viewing patterns (Glashouwer et al., 2016), even though body dissatisfaction improved. This finding, in particular, questions whether change in attention is an important mechanism underlying the effects of ME. As no clinical groups were examined in Glashouwer et al. (2016), floor effects could have contributed to these results (Naumann et al., 2022).

Thus, the question remains whether dysfunctional attentional processes are maintaining mechanisms of body dissatisfaction and whether reducing this bias is a working mechanism of action of ME. Establishing whether changing biased attention towards the own body is a mechanism underlying the effects of ME in reducing body dissatisfaction in people with AN is therefore an important research target. In the present study, eye-tracking data, more specifically, tracking gaze on body stimuli, which has been successfully used in body image research as an objective measure of attentional bias (Bauer et al., 2017; Blechert et al., 2010; Jansen et al., 2005), is used to test whether the assumed selective gaze pattern of patients with AN on unattractive body parts can be successfully modified by a mirror exposure intervention, leading to a reduction in body dissatisfaction.

In a study of Bauer et al. (2017) all eating disorder subgroups had an attentive preference for body areas they find unattractive, with even longer fixation time on self-evaluated unattractive areas of one's own body compared to fixation time on the body of peer's. Participants with AN-R attended significantly longer to unattractive body areas in general and significantly shorter to attractive areas than the control groups (clinical control group with anxiety disorder and healthy controls). Therefore, we aim to investigate attention bias in different variations (single presentation vs. simultaneous presentation of own/other bodies as well as neutral stimuli), analyzing the areas of the bodies (most unattractive/attractive) to which the participants allocate their visual attention. Furthermore, we collect and analyze reaction times as an indirect measure of attentional biases through the cueing and dot-probe task (MacLeod et al., 1986; Posner, 1980).

## Aims

The main aim of this randomized controlled trial is to test the efficacy of ME in adolescents with AN. We expect that ME significantly reduces body dissatisfaction compared to a waitlist control group. In addition, we will examine whether the change in body dissatisfaction relates to a reduction in the general eating disorder psychopathology. In exploratory analyses, we also aim to examine whether ME compared to a waitlist control group leads to a change of the behavioral components of body image disturbance (body checking and body avoidance).

Secondly, we expect that ME compared to a waitlist control group leads to a stronger reduction of body-related attention bias (pre-post comparison). Finally, we expect that changes in body-related attention bias are associated with changes in body dissatisfaction, body-related emotions and cognitions. Additionally, we want to explore possible process variables and predictors of treatment success.

## Method

### Trial Design

**Figure 1:**
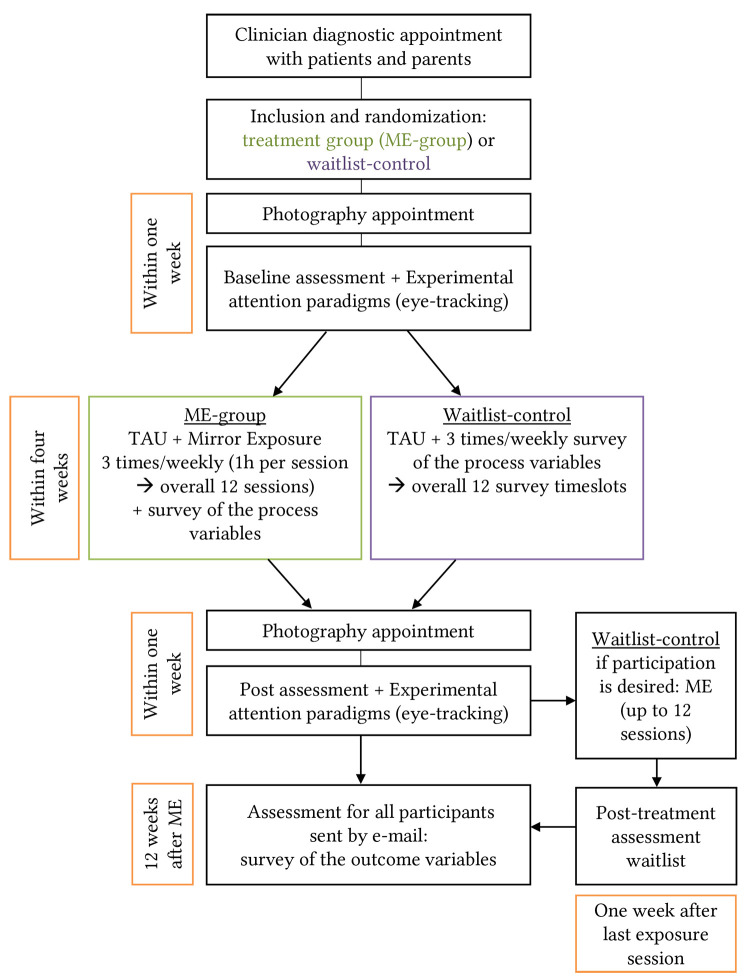
Trial Design

This feasibility study is designed as RCT (experimental: ME, control: waitlist) with pre- and post-comparison (6 weeks) and open follow-up (12 weeks). Participants are randomly allocated to receive either 12 sessions of ME (treatment group) in addition to TAU or to waitlist (control group), who will receive TAU only. TAU includes behavioral therapy interventions and nutrition management according to the German S3 Guideline for Diagnosis and Treatment of eating disorders. More detail regarding the randomization procedure is provided below. The study design is shown in Figure 1.

### Ethical Approval and Trial Registration

Ethical approval has been obtained from the Ethics Committee of the Albert-Ludwigs-University in Freiburg, Germany (545/17). Participants received verbal and written (consent) information before participating. In the case of underage participants, their legal guardians are also informed and their consent to participate is obtained as well. The research is conducted in accordance with the Declaration of Helsinki. The study is registered on the German Clinical Trial Register (DRKS; registration number: DRKS0019104).

### Participants and Recruitment

Participants are recruited via the Department of Child and Adolescent Psychiatry, Psychotherapy and Psychosomatics of the Freiburg University Hospital (director: Prof. Dr. Fleischhaker) and from the outpatient unit of the Institute of Psychology, Department of Clinical Psychology and Psychotherapy, University of Freiburg (head: Prof. Dr. Tuschen-Caffier).

### Inclusion Criteria

Girls and young women are eligible for participating if they are diagnosed with an ICD-10 of AN or atypical AN, age > 12 < 21. In addition, participants are not allowed to be currently tube fed and their weight must be above the 10th BMI percentile at the time-point of inclusion.

### Exclusion Criteria

Exclusion criteria are high risk of suicide, co-occurring psychotic, bi-polar disorders, alcohol/substance dependence within the past six months, medical conditions that would affect the ability to participate, and pregnancy/lactation.

### ME Procedure and Waitlist Control Group

The ME technique applied in the current study is based on the manual of Hilbert and Tuschen-Caffier (2010) as well as on the ME protocol used in Trentowska et al. (2013). ME sessions are delivered in an individual setting ﻿in standardized underwear (beige/ white panty and top) by one of two post-graduate psychologists, who are enrolled in clinical CBT-training to become a licensed therapist.

Participants are instructed to look in a full-length mirror with double winged doors. The therapist stands outside the participant’s view and asks her to describe her own body as precisely as possible from head to toe. During the ME, participants can freely express their positive and negative feelings and the therapist encourages the participant to name and persevere any upcoming feelings as part of the exposure rationale. ME sessions lasts 50-60 min and followed by a brief debriefing.

The waitlist control group receives TAU. TAU entails integral CBT-based treatment about nutrition management, eating behavior, stress management, social competence training and body-image treatment. However, participants in the waitlist control retain from receiving any body-image related treatment during the four weeks of study participation to prevent confounding of ME effects.

### Therapist Training and Supervision

All ME therapists received an introduction to the ME rationale and treatment manual as well as ME practice sessions within the research group before starting with ME in the study. Regular supervision is provided to therapists by senior ME therapists. To ensure treatment adherence, ME sessions are video recorded and will be discussed during supervision. These are deleted after each supervision.

### Outcome Measures

Since this is a feasibility study, a broad range of outcome measures is included to determine which are most sensitive for detecting a treatment effect. Table 1 provides an overview of outcome measurements.

**Table 1:** Schedule of Assessments

Content	Assessment	Screening	Baseline	Within-session	Post	Post-treatment^a^	After 3 months
Participant’s information, informed consent		x					
Clinical and demographic information		x					
ChEDE		x					
K-SADS-PL		x					
Affective and cognitive components of body image disturbances	FFB		x		x	x	x
Behavioral components of body image disturbances	BCQ		x		x	x	x
	BIAQ		x		x	x	x
Attention-bias	exogenus cueing paradigm		x		x		
	adapted version of the dot probe task		x		x		
Mood and body evaluation over course of ME	PANAS		x	x	x	x	x
	“Body-Questionnaire”		x	x	x		
others	BDI-II		x		x	x	x
	RSE		x		x	x	x
questions about the conditions						x	x
treatment evaluation							x

*Note.* BDI-II = Beck Depression Inventory; BCQ = Body Checking Questionnaire; BIAQ = Body Image Avoidance Questionnaire; ChEDE = Eating Disorder Examination for children; ChEDE-Q = Child Eating Disorder Examination Questionnaire; FFB = Fragebogen zum Figurbewusstsein; K-SADS-PL = Kiddie Schedule for Affective Disorders and Schizophrenia Lifetime Version; PANAS = Positive and Negative Affect Scales; RSE = Rosenberg Self-Esteem Scale.

^a^Only for waitlist control who received ME.

#### Primary Outcome

##### Affective and Cognitive Components of Body Image Disturbances

To assess body dissatisfaction, as affective-cognitive component of body image disturbance, the German version of the Body Shape Questionnaire (BSQ; Cooper et al., 1987; German version: Fragebogen zum Figurbewusstsein, FFB; Waadt et al., 1992) is used. The FFB includes 34 items and is a widely used measurement tool to record numerous aspects of dissatisfaction with one's body shape with good psychometric properties (Pook et al., 2002).

##### Behavioral Components of Body Image Disturbances

Exploratory, behavioral components of body image disturbances are assessed with the Body Checking Questionnaire (BCQ; Reas et al., 2002; German version: Vocks et al., 2008) and the Body Image Avoidance Questionnaire (BIAQ; Rosen et al., 1991; German version: Legenbauer et al., 2007). The BCQ is a 23-item reliable and valid instrument for assessing body-related control behavior (Steinfeld et al., 2017). The BIAQ is a 19-item self-assessment tool for body-related avoidance and eating-related control behaviors with good psychometric properties (Legenbauer et al., 2007).

##### General Eating Disorder Pathology

ED symptomatology will be measured by the German version of the Child Eating Disorder Examination Questionnaire (ChEDE-Q; TODAY Study Group, 2007; German version: Hilbert et al., 2008). This child version of the Eating Disorder Examination-Questionnaire by Fairburn and Beglin (EDE-Q, 1994, 2008; German-version: Hilbert & Tuschen-Caffier, 2006, 2016) allows the assessment of the specific eating disorder psychopathology on four subscales (Restraint, Eating Concern, Weight Concern and Shape Concern) with 28 items. The German translation of the ChEDE-Q proved to be good internal consistency, convergent validity and retest reliability over a period of 7.5 months (Hilbert et al., 2008).

#### Secondary Outcomes

##### Attention Bias

Attention bias will comprise eye-tracking based attention processing of individually self-defined unattractive versus attractive body parts. Two attention paradigms (exogenous cueing paradigm and dot probe paradigm) are used to assess body-related attention biases. Both paradigms rely on the assessment of eye-tracking to index overt spatial attention allocation to body stimuli.

##### Stimulus Material

In both tasks, standardized photographs of the participants’ own body and a control body matched in BMI and waist-to-hip ratio are used as body stimuli. Vases are used as neutral/non-body-related control stimuli (Krohmer et al., 2022b). Participants wear standardized underwear (beige/ white panty and top) and are photographed in standardized positions (hip wide stand, arms beside the body, back of the hands forward with fingers extended) from four perspectives (front, left, right, back) without the face and feet being visible. The photos are transferred in black and white and presented on a gray background. Noticeable features (tattoos, scars) are removed.

##### Body-Related Exogenous Cueing Paradigm

In the exogenous cueing paradigm participants view their own body or a weight-matched control body on one side of the screen (either left or right) for 3000 ms and need to indicate the location of a cue appearing subsequently on either the left or right side of the screen (valid or invalid with the body’s position). ﻿ Accordingly, the paradigm consists of the following trial types: own body and other body in four perspectives (front, left, right, back) presented on right/left side with valid/ invalid cue = 32 trials, repeated in 4 blocks = 128 trials in total. The bias scores indexes attention allocation towards self-defined attractive and unattractive body parts of the own versus the other body without a direct competing stimulus. Frequency and duration of fixations on areas of interest (self-rated attractive vs. self-rated unattractive body parts of the own and the other body) are extracted for further analyses.

##### Body-Related Version of the Dot Probe Task

In this adapted version of the dot probe participants view stimulus pairs for 3000 ms and need to indicate the location of a cue appearing subsequently on either the left or right side of the screen, replacing one of the two stimuli. The following trial types are presented as stimulus pairs: own body/vase, other body/vase, own body/other body (each pair in 4 perspectives), presented on right/left side with valid/ invalid cue = 48 trials, repeated in 2 blocks = 96 trials in total. Gaze pattern during the presentation of these picture pairs indexes attention allocation towards the own body versus other body when a competing stimulus is presented at the same time. The bias scores of this paradigm indexes attention allocation towards own versus a direct competing neutral or another body stimulus. The frequency of the direction and the duration of the first and second fixation towards the own body when compared to a neutral stimulus or another body will be analyzed.

##### Mood and Body Evaluation Over Course of ME

To explore other potential processes of change during ME, we assess mood and the evaluation of one's own body over the course of ME. Mood is assessed using the German Version of the Positive and Negative Affect Scales (PANAS; Watson et al., 1988; German version: Krohne et al., 1996), which consists of 20 adjectives that describe different sensations and feelings (10 positive, 10 negative feelings). The German PANAS has very good psychometric properties (Breyer & Bluemke, 2016). The evaluation of one's own body is rated with the ‘Body Questionnaire’ (see e.g. Tuschen-Caffier et al., 2015), which assesses state body dissatisfaction and obtains attractiveness ratings of specific body parts based on photographs of participants as used in the attention paradigms.

##### Sample Characteristics

Age and duration of illness are measured. To assess severity of depression, the Beck Depression Inventory (BDI-II; Beck et al., 1996; German version: Hautzinger et al., 2006) is used, which is a 21-items self-report instrument for the severity of depressive mood over the last two weeks with high validity and reliability (Keller et al., 2022). Because self-esteem has been linked to body dissatisfaction and ED- symptoms, we assess self-esteem at baseline validly and reliably using the 10-item Rosenberg Self-Esteem Scale (RSE; Rosenberg, 1965); German version: von Collani & Herzberg, 2003).

### Procedure

Potential participants are referred to the study by their clinician/therapists. Study researchers screen participants for eligibility. Once eligibility has been established, patient’s and their parents’, in the case of underage patients, written informed consents are obtained. Eligible participants are invited for a diagnostic session. Eating disorder diagnoses are established by means of the German version of the ChEDE (Bryant-Waugh et al., 1996; Hilbert, 2016). Other mental disorder diagnoses are assessed by means of the German version of the Kiddie Schedule for Affective Disorders and Schizophrenia Lifetime Version (K-SADS-PL; Delmo et al., 2001; Kaufman et al., 2000). If participants meet all inclusion criteria, participants are randomly allocated to the treatment or waitlist control group. Within one week, the photo appointment takes place, to create four standardized photos of participants, which are used as stimulus material in the attention paradigms.

Then, participants are invited to the baseline assessment, in which they complete the eye tracking paradigms and questionnaires on relevant outcome measures. If allocated to the ME condition, an initial session to explain the rationale and procedure of ME is scheduled first, followed by three sessions of ME per week for four weeks. Directly after each ME session, participants answer questionnaires assessing their body dissatisfaction and mood. In the waitlist control, participants also have three appointments per week, during which only body dissatisfaction and mood are assessed. After completing 12 sessions of ME or waitlist appointments, respectively, the post assessment takes place in the week following the last session. This outcome assessment of eye-tracking paradigms and questionnaires is identical to the baseline assessment. Participants in the waitlist control group are offered ME (at their own convenience) after completing post-treatment assessments.

Three months after the post assessment, participants who were allocated to the ME group receive an email containing a link to an online-questionnaire asked about the individually experiences and evaluation of ME regarding their subjective experiences of acceptability, satisfaction and recommendation. Participants from the waitlist control group who took up ME after post assessment evaluate their experience one week after their last ME session and again after three months.

### Randomization

Before the start of the study, a randomization list was prepared by the project management. To ensure blinding during screening and diagnostics, the project management informs the researcher and the therapists on condition allocation only after inclusion of a patient.

### Sample Size and Current Trial Status

Sample size calculation yielded with a power of (1-β) = .80, a moderate to large effect (Cohen, 1988), based on previous results (Key et al., 2002; Morgan et al., 2014), of *d* = 0.8–1.3, α = .05 and a moderate correlation of within-effects the sample size – calculated over generic tests – at least around 42 patients with AN should be included. The study was initiated in September 2018. By January 2023, 24 patients have participated. The study recruitment has been repeatedly interrupted for various reasons (e.g., the Corona pandemic). With a study participation of 1-2 patients per month so far, the study is expected to run until December 2023.

### Statistical Analysis

To determine quality, completeness and variability of the outcome measures, descriptive statistical analyses and graphical methods will be used. To test if ME significantly reduces body dissatisfaction compared to waitlist control group (first hypothesis) 2 x 2 one-way analyses of variance (ANOVA) with group (ME/waitlist) as between- subject factor, time (pre/post) as within-subject factor will be applied. In exploratory analyses we also aim to test if ME significantly reduces body checking and body avoidance compared to waitlist control group 2 x 2 one-way analyses of variance (ANOVA) with group (ME/waitlist) as between- subject factor, time (pre/post) as within-subject factor will be applied. For our secondary hypothesis (i.e. ME compared to wait-list results in reductions of body-related attention bias), a mixed 2 (group: ME/ waitlist) x 2 (time: pre/post) x 2 (stimulus material: self/other) x 2 (body party: unattractive/attractive) ANOVA for both attention paradigms is planned. We will define areas of interest (AOI) based on participants` ratings of the most attractive and unattractive body part (for own/other body respectively). Bias scores for gaze duration, gaze frequency and number of initial fixations on each stimulus will serve as dependent variables. To clarify if change in attention bias is associated with change in body dissatisfaction, correlations between (changes) of attentional biases and body dissatisfaction, body checking and body avoidance will be conducted.

In exploratory analyses we also aim to capture processes of change in mood and body evaluation over the course of repeated ME sessions and how potential change in these measures relates to changes in relevant outcome variables such as body dissatisfaction, attention bias and global ED pathology. Finally, we will also explore how possible participants’ characteristics, such as age or self-esteem, relate to improvements in body dissatisfaction after ME.

## Discussion

Dissatisfaction with one's own body is a major risk factor for the development, maintenance, and relapse of AN (Glashouwer et al., 2019; Jacobi et al., 2004). ME is an effective technique for treating body dissatisfaction in adults (Ziser et al., 2018). However, there is limited research on the effectiveness of ME therapy in AN, and even less research has been conducted on the effects of ME in children and adolescents with AN (Biney et al., 2021). The aim of this study is to address this research gap by examining the effects of ME on body dissatisfaction in children and adolescents with AN. The secondary aim is to clarify whether the change in attentional bias for body stimuli is the mechanism underlying the change in body dissatisfaction due to ME. In this feasibility RCT we will also strive to explore additional variables of interest, such as body-related emotions and cognitions.

### Strengths

A strength of this study is conducting experimental psychopathology research in the field of adolescents with AN and in residential facilities because this is particularly difficult context for experimental studies (Glashouwer et al., 2020). Considering the high prevalence of AN among adolescents and minimal treatment effects in the treatment of AN (e.g. Zeeck et al., 2018), we know how important it is to conduct experimental research to test novel treatment options and to study working mechanisms of current treatment techniques as well as mechanisms contributing to the maintenance of AN. The present study achieves a greater understanding of ME as treatment technique for adolescents with AN as well as providing initial evidence for a potential working mechanisms of this technique (change in body-related attention bias) and maintaining factor of body dissatisfaction in this sample (i.e. dysfunctional body-related attention patterns). In addition, this experimental study conducted as an add-on to TAU offers the opportunity for patients to participate in and benefit from this treatment technique (ME). Another strength of the current study is combining a feasibility RCT in this context with multimethodological outcome assessment, including the direct assessment of overt spatial attention allocation by means of eye-tracking. This multimethod approach can inform on subjective as well as relatively automatic cognitive changes due to the treatment (ME). Eye-tracking has been established as a valid instrument to index visual attention processing (Blechert et al., 2009; van Ens et al., 2019).

### Challenges

One major challenge remains consistent recruitment – even though there is the high number of people affected by AN in adolescence and in particular the prevalence of AN in adolescents amid the COVID epidemic and the demand for therapy are increasing. Consistent recruitment may also be challenging because facilitating research in an inpatient clinic during a pandemic has led to disruptions in concurrent recruitment procedures. In addition, the integration of a study in a clinical setting with a highly intensive therapy program for patients, is particularly challenging in the clinical context regarding logistics as well as time-planning organization.

### Conclusion

To conclude, this paper sets out a protocol for an RCT that will enhance the current knowledge of the efficacy of ME to target body dissatisfaction as central core symptom for AN in adolescents.

## Study Registration

The study is registered on the German Clinical Trial Register (DRKS; registration number: DRKS0019104).

## References

[r1] Alleva, J. M., Sheeran, P., Webb, T. L., Martijn, C., & Miles, E. (2015). A meta-analytic review of stand-alone interventions to improve body image. PLoS One, 10(9), e0139177. 10.1371/journal.pone.0139177 26418470 PMC4587797

[r2] Bauer, A., Schneider, S., Waldorf, M., Braks, K., Huber, T. J., Adolph, D., & Vocks, S. (2017). Selective visual attention towards oneself and associated state body satisfaction: An eye-tracking study in adolescents with different types of eating disorders. Journal of Abnormal Child Psychology, 45(8), 1647–1661. 10.1007/s10802-017-0263-z 28133705

[r3] Beck, A. T., Steer, R. A., & Brown, G. K. (1996). *Manual for the Beck Depression Inventory – II.* Psychological Corporation.

[r4] Berman, M. I., Boutelle, K. N., & Crow, S. J. (2009). A case series investigating acceptance and commitment therapy as a treatment for previously treated, unremitted patients with anorexia nervosa. European Eating Disorders Review, 17(6), 426–434. 10.1002/erv.962 19760625

[r5] Bhatnagar, K. A. C., Wisniewski, L., Solomon, M., & Heinberg, L. (2013). Effectiveness and feasibility of a cognitive-behavioral group intervention for body image disturbance in women with eating disorders. Journal of Clinical Psychology, 69(1), 1–13. 10.1002/jclp.21909 22903360

[r6] Biney, H., Astbury, S., Haines, A., Grant, J., Malone, N., Hutt, M., Matthews, R., Morgan, J. F., White, S., & Lacey, J. H. (2021). A novel ‘practical body image’ therapy for adolescent inpatients with anorexia nervosa: A randomised controlled trial. Eating and Weight Disorders, 26(6), 1825–1834. 10.1007/s40519-020-00997-2 32949382 PMC8292282

[r7] Blechert, J., Ansorge, U., & Tuschen-Caffier, B. (2010). A body-related dot-probe task reveals distinct attentional patterns for bulimia nervosa and anorexia nervosa. Journal of Abnormal Psychology, 119(3), 575–585. 10.1037/a0019531 20677846

[r8] Blechert, J., Nickert, T., Caffier, D., & Tuschen-Caffier, B. (2009). Social comparison and its relation to body dissatisfaction in bulimia nervosa: Evidence from eye movements. Psychosomatic Medicine, 71(8), 907–912. 10.1097/PSY.0b013e3181b4434d 19661192

[r9] Breyer, B., & Bluemke, M. (2016). Deutsche Version der Positive and Negative Affect Schedule PANAS (GESIS Panel) [German version of the positive and negative affect schedule PANAS]. *Zusammenstellung sozialwissenschaftlicher Items und Skalen (ZIS)*. 10.6102/zis242

[r10] Brockmeyer, T., Friederich, H. C., & Schmidt, U. (2018). Advances in the treatment of anorexia nervosa: A review of established and emerging interventions. Psychological Medicine, 48(8), 1228–1256. 10.1017/S0033291717002604 28889819

[r11] Bryant-Waugh, R. J., Cooper, P. J., Taylor, C. L., & Lask, B. D. (1996). The use of the eating disorder examination with children: A pilot study. International Journal of Eating Disorders, 19(4), 391–397. 10.1002/(SICI)1098-108X(199605)19:4<391::AID-EAT6>3.0.CO;2-G 8859397

[r12] Cohen, J. (1988). *Statistical power analysis for the behavioral sciences* (2nd ed.). Erlbaum.

[r13] Cooper, P. J., Taylor, M. J., Cooper, Z., & Fairbum, C. G. (1987). The development and validation of the Body Shape Questionnaire. International Journal of Eating Disorders, 6(4), 485–494. 10.1002/1098-108X(198707)6:4<485::AID-EAT2260060405>3.0.CO;2-O

[r14] Delmo, C., Weiffenbach, O., Gabriel, M., Stadler, C., & Poustka, F. (2001). *Diagnostic Interview Kiddie-Sads-Present and Lifetime Version (K-SADS-PL). 5. Auflage der deutschen Forschungsversion, erweitert um ICD-10-Diagnostik* [Diagnostic Interview Kiddie-Sads-Present and Lifetime Version (K-SADS-PL). 5th edition of the German research version, expanded to include ICD-10 diagnosis]. http://www.adhs-essen.com/PDF/K-SADS_Fragebogen.pdf

[r15] DuBois, R. H., Rodgers, R. F., Franko, D. L., Eddy, K. T., & Thomas, J. J. (2017). A network analysis investigation of the cognitive-behavioral theory of eating disorders. Behaviour Research and Therapy, 97, 213–221. 10.1016/j.brat.2017.08.004 28826067

[r16] Fairburn, C. G., & Beglin, S. J. (1994). *Eating Disorder Examination Questionnaire (EDE-Q)* [Database record]. APA PsycTests. 10.1037/t03974-000

[r17] Fairburn, C. G., & Beglin, S. J. (2008). Eating Disorder Examination Questionnaire (EDE–Q 6.0). In *Cognitive behavior therapy and eating disorders* (pp. 309-313). Guilford Press.

[r18] Forrest, L. N., Jones, P. J., Ortiz, S. N., & Smith, A. R. (2018). Core psychopathology in anorexia nervosa and bulimia nervosa: A network analysis. International Journal of Eating Disorders, 51(7), 668–679. 10.1002/eat.22871 29693747

[r69] Glashouwer, K. A., Brockmeyer, T., Cardi, V., Jansen, A., Murray, S. B., Blechert, J., Levinson, C. A., Schmidt, U., Tchanturia, K., Wade, T. D., Svaldi, J., Giel, K. E., Favaro, A., Fernández-Aranda, F., Friederich, H.-C., Naumann, E., Treasure, J. L., Tuschen-Caffier, B., Vocks, S., & Werthmann, J. (2020). Time to make a change: A call for more experimental research on key mechanisms in anorexia nervosa. European Eating Disorders Review, 28(4), 361–367. 10.1002/erv.2754 32567176

[r19] Glashouwer, K. A., Jonker, N. C., Thomassen, K., & de Jong, P. J. (2016). Take a look at the bright side: Effects of positive body exposure on selective visual attention in women with high body dissatisfaction. Behaviour Research and Therapy, 83, 19–25. 10.1016/j.brat.2016.05.006 27236075

[r20] Glashouwer, K. A., van der Veer, R. M. L., Adipatria, F., de Jong, P. J., & Vocks, S. (2019). The role of body image disturbance in the onset, maintenance, and relapse of anorexia nervosa: A systematic review. Clinical Psychology Review, 74, 101771. 10.1016/j.cpr.2019.101771 31751876

[r21] Griffen, T. C., Naumann, E., & Hildebrandt, T. (2018). Mirror exposure therapy for body image disturbances and eating disorders: A review. Clinical Psychology Review, 65, 163–174. 10.1016/j.cpr.2018.08.006 30223161

[r22] Grilo, C. M., & Udo, T. (2021). Examining the significance of age of onset in persons with lifetime anorexia nervosa: Comparing child, adolescent, and emerging adult onsets in nationally representative U.S. study. International Journal of Eating Disorders, 54(9), 1632–1640. 10.1002/eat.23580 34263464 PMC8416938

[r23] Hartmann, A. S., Naumann, E., Vocks, S., Svaldi, J., & Werthmann, J. (2021). Body exposure, its forms of delivery and potentially associated working mechanisms: How to move the field forward. Clinical Psychology in Europe, 3(3), e3813. 10.32872/cpe.3813 36398104 PMC9667231

[r24] Hautzinger, M., Keller, F., & Kühner, C. (2006). *BDI-II. Beck‐Depressions‐Inventar Revision – Manual.* Harcourt Test Services.

[r25] Herpertz-Dahlmann, B., Dempfle, A., & Eckardt, S. (2022). The youngest are hit hardest: The influence of the COVID-19 pandemic on the hospitalization rate for children, adolescents, and young adults with anorexia nervosa in a large German representative sample. European Psychiatry, 65(1), e84. 10.1192/j.eurpsy.2022.2345 36403977 PMC9748980

[r26] Hilbert, A. (2016). *Eating Disorder Examination für Kinder: Deutschsprachige Übersetzung* [Eating Disorder Examination for children: German translation]. dgvt-Verlag.

[r27] Hilbert, A., Hartmann, A. S., & Czaja, J. (2008). Child Eating Disorder Examination-Questionnaire: Psychometrische Eigenschaften der deutschsprachigen Übersetzung [Child eating disorder examination-questionnaire: Psychometric characteristics of the German version]. Klinische Diagnostik und Evaluation, 1, 447–463.

[r28] Hilbert, A., & Tuschen-Caffier, B. (2006). *Eating Disorder Examination – Questionnaire: Deutschsprachige Übersetzung* (Bd. 2). Verlag für Psychotherapie.

[r29] Hilbert, A., & Tuschen-Caffier, B. (2010). *Essanfälle und Adipositas: Ein Manual zur kognitiv-behavioralen Therapie der Binge-Eating Störung* [Binge eating and obesity: A manual of cognitive behavioral therapy for binge eating disorder]. Hogrefe.

[r30] Hilbert, A., & Tuschen-Caffier, B. (2016). *Eating Disorder Examination-Questionnaire: Deutschsprachige Übersetzung* (Bd. 2, 2. Auflage). dgvt-Verlag.

[r31] Jacobi, C., Hayward, C., de Zwaan, M., Kraemer, H. C., & Agras, W. S. (2004). Coming to terms with risk factors for eating disorders: Application of risk terminology and suggestions for a general taxonomy. Psychological Bulletin, 130(1), 19–65. 10.1037/0033-2909.130.1.19 14717649

[r32] Jansen, A., Nederkoorn, C., & Mulkens, S. (2005). Selective visual attention for ugly and beautiful body parts in eating disorders. Behaviour Research and Therapy, 43(2), 183–196. 10.1016/j.brat.2004.01.003 15629749

[r33] Jansingh, A., Danner, U. N., Hoek, H. W., & van Elburg, A. A. (2020). Developments in the psychological treatment of anorexia nervosa and their implications for daily practice. Current Opinion in Psychiatry, 33(6), 534–541. 10.1097/YCO.0000000000000642 32796187 PMC7575018

[r34] Jiang, M. Y. W., & Vartanian, L. R. (2018). A review of existing measures of attentional biases in body image and eating disorders research. Australian Journal of Psychology, 70(1), 3–17. 10.1111/ajpy.12161

[r35] Kaufman, J., Birmaher, B., Brent, D. A., Ryan, N. D., & Rao, U. (2000). K-SADS-PL. Journal of the American Academy of Child and Adolescent Psychiatry, 39(10), 1208. 10.1097/00004583-200010000-00002 11026169

[r36] Keller, F., Kühner, C., Alexandrowicz, R. W., Voderholzer, U., Meule, A., Fegert, J. M., Legenbauer, T., Holtmann, M., Bräscher, A.-K., Cordes, M., Fehm, L., Fladung, A.-K., Fydrich, T., Hamm, A., Heider, J., Hoyer, J., In-Albon, T., Lincoln, T. M., Lutz, W., . . . Hautzinger, M. (2022). Zur Messqualität des Beck-Depressionsinventars (BDI-II) in unterschiedlichen klinischen Stichproben [The evaluation of the Beck Depression Inventory (BDI-II) in different clinical samples]. Zeitschrift für Klinische Psychologie und Psychotherapie, 51(3-4), 234–246. 10.1026/1616-3443/a000676

[r37] Kerr-Gaffney, J., Harrison, A., & Tchanturia, K. (2019). Eye-tracking research in eating disorders: A systematic review. International Journal of Eating Disorders, 52(1), 3–27. 10.1002/eat.22998 30582199

[r38] Key, A., George, C. L., Beattie, D., Stammers, K., Lacey, H., & Waller, G. (2002). Body image treatment within an inpatient program for anorexia nervosa: The role of mirror exposure in the desensitization process. International Journal of Eating Disorders, 31(2), 185–190. 10.1002/eat.10027 11920979

[r39] Krohmer, K., Naumann, E., Tuschen-Caffier, B., & Svaldi, J. (2022 a). Mirror exposure in binge-eating disorder: Changes in eating pathology and attentional biases. Journal of Consulting and Clinical Psychology, 90(8), 613–625. 10.1037/ccp0000751 36066863

[r40] Krohmer, K., Naumann, E., Tuschen-Caffier, B., & Svaldi, J. (2022 b). Taking a closer look at body processing in binge eating disorder – Influence of BMI and eating pathology. Behaviour Research and Therapy, 156, 104106. 10.1016/j.brat.2022.104106 35724597

[r41] Krohne, H. W., Egloff, B., Kohlmann, C.-W., & Tausch, A. (1996). *Positive and Negative Affect Schedule – German Version (PANAS)* [Database record]. APA PsycTests. 10.1037/t49650-000

[r42] Legenbauer, T., Vocks, S., & Schütt-Strömel, S. (2007). Validierung einer Deutschsprachigen Version des Body Image Avoidance Questionnaire BIAQ [Validation of a German version of the Body Image Avoidance Questionnaire BIAQ]. Diagnostica, 53(4), 218–225. 10.1026/0012-1924.53.4.218

[r43] MacLeod, C., Mathews, A., & Tata, P. (1986). Attentional bias in emotional disorders. Journal of Abnormal Psychology, 95(1), 15–20. 10.1037/0021-843X.95.1.15 3700842

[r44] Mitchison, D., Rieger, E., Harrison, C., Murray, S. B., Griffiths, S., & Mond, J. (2018). Indicators of clinical significance among women in the community with binge-eating disorder symptoms: Delineating the roles of binge frequency, body mass index, and overvaluation. International Journal of Eating Disorders, 51(2), 165–169. 10.1002/eat.22812 29278426

[r45] Morgan, J. F., Lazarova, S., Schelhase, M., & Saeidi, S. (2014). Ten session body image therapy: Efficacy of a manualised body image therapy. European Eating Disorders Review, 22(1), 66–71. 10.1002/erv.2249 24006359

[r46] Naumann, E., Werthmann, J., Vocks, S., Svaldi, J., & Hartmann, A. S. (2022). Die Spiegelkonfrontationstherapie zur Behandlung von Körperbildstörungen bei Essstörungen – Evidenz, Wirkmechanismen und Vorgehensweise [Mirror exposure therapy for the treatment of body image disorders in eating disorders – Evidence, mechanisms of action and procedure]. Psychologische Rundschau, 73(4), 243–259. 10.1026/0033-3042/a000558

[r47] Nestoriuc, Y., Berking, M., & Rief, W. (2012). Psychotherapieforschung [Psychotherapy research]. In M. Berking & W. Rief (Eds.), *Klinische Psychologie und Psychotherapie für Bachelor* [Clinical Psychology and Psychotherapy for Bachelor] (pp. 165–179). Springer.

[r48] Pook, M., Tuschen-Caffier, B., & Stich, N. (2002). Evaluation des Fragebogens zum Figurbewusstsein (FFB, deutsche Version des Body Shape Questionnaire) [Evaluation of the German version of the Body Shape Questionnaire]. Verhaltenstherapie, 12(2), 116–124. 10.1159/000064375

[r49] Posner, M. I. (1980). Orienting of attention. The Quarterly Journal of Experimental Psychology, 32(1), 3–25. 10.1080/00335558008248231 7367577

[r50] Reas, D. L., Whisenhunt, B. L., Netemeyer, R., & Williamson, D. A. (2002). Development of the body checking questionnaire: A self-report measure of body checking behaviors. International Journal of Eating Disorders, 31(3), 324–333. 10.1002/eat.10012 11920995

[r51] Rodgers, R. F., & DuBois, R. H. (2016). Cognitive biases to appearance-related stimuli in body dissatisfaction: A systematic review. Clinical Psychology Review, 46, 1–11. 10.1016/j.cpr.2016.04.006 27116714

[r52] Rosen, J. C., Srebnik, D., Saltzberg, E., & Wendt, S. (1991). Development of a body image avoidance questionnaire. Psychological Assessment, 3(1), 32–37. 10.1037/1040-3590.3.1.32

[r53] Rosenberg, M. (1965). *Rosenberg Self-Esteem Scale (RSES)* [Database record]. APA PsycTests. 10.1037/t01038-000

[r54] Smeets, E., Jansen, A., & Roefs, A. (2011). Bias for the (un)attractive self: On the role of attention in causing body (dis)satisfaction. Health Psychology, 30(3), 360–367. 10.1037/a0022095 21553980

[r55] Steinfeld, B., Bauer, A., Waldorf, M., Engel, N., Braks, K., Huber, T. J., & Vocks, S. (2017). Validierung einer deutschsprachigen Fassung des Body Checking Questionnaire (BCQ) an Jugendlichen mit Anorexia und Bulimia Nervosa [Validation of a German-language version of the Body Checking Questionnaire (BCQ) in adolescents with anorexia and bulimia nervosa]. Psychotherapie, Psychosomatik, Medizinische Psychologie, 67(1), 38–46. 10.1055/s-0042-110851 27711955

[r56] Stice, E., Marti, C. N., & Rohde, P. (2013). Prevalence, incidence, impairment, and course of the proposed DSM-5 eating disorder diagnoses in an 8-year prospective community study of young women. Journal of Abnormal Psychology, 122(2), 445–457. 10.1037/a0030679 23148784 PMC3980846

[r57] Stice, E., Marti, C. N., Shaw, H., & Jaconis, M. (2009). An 8-year longitudinal study of the natural history of threshold, subthreshold, and partial eating disorders from a community sample of adolescents. Journal of Abnormal Psychology, 118(3), 587–597. 10.1037/a0016481 19685955 PMC2849679

[r58] TODAY Study Group. (2007). Treatment options for type 2 diabetes in adolescent and youth: A study of the comparative efficacy of metformin alone or in combination with rosiglitazone or lifestyle intervention in adolescents with type 2 diabetes. Pediatric Diabetes, 8(2), 74–87. 10.1111/j.1399-5448.2007.00237.x 17448130 PMC2752327

[r59] Trentowska, M., Bender, C., & Tuschen-Caffier, B. (2013). Mirror exposure in women with bulimic symptoms: How do thoughts and emotions change in body image treatment? Behaviour Research and Therapy, 51(1), 1–6. 10.1016/j.brat.2012.03.012 23168326

[r60] Tuschen-Caffier, B., Bender, C., Caffier, D., Klenner, K., Braks, K., & Svaldi, J. (2015). Selective visual attention during mirror exposure in anorexia and bulimia nervosa. PLoS One, 10(12), e0145886. 10.1371/journal.pone.0145886 26714279 PMC4700997

[r61] van Ens, W., Schmidt, U., Campbell, I. C., Roefs, A., & Werthmann, J. (2019). Test-retest reliability of attention bias for food: Robust eye-tracking and reaction time indices. Appetite, 136, 86–92. 10.1016/j.appet.2019.01.020 30682381

[r62] Vocks, S., Busch, M., Schulte, D., Grönermeyer, D., Herpertz, S., & Suchan, B. (2010). Effects of body image therapy on the activation of the extrastriate body area in anorexia nervosa: An fMRI study. Psychiatry Research: Neuroimaging, 183(2), 114–118. 10.1016/j.pscychresns.2010.05.011 20630712

[r63] Vocks, S., Wächter, A., Wucherer, M., & Kosfelder, J. (2008). Look at yourself: Can body image therapy affect the cognitive and emotional response to seeing oneself in the mirror in eating disorders? European Eating Disorders Review, 16(2), 147–154. 10.1002/erv.825 17721910

[r64] von Collani, G., & Herzberg, P. Y. (2003). Eine revidierte Fassung der deutschsprachigen Skala zum Selbstwertgefühl von Rosenberg [A revised version of the German version of Rosenberg's Self-Esteem Scale]. Zeitschrift für Differentielle und Diagnostische Psychologie, 24(1), 3–7. 10.1024//0170-1789.24.1.3

[r65] Waadt, S., Laessle, R. G., & Pirke, K. M. (1992). *Bulimie. Ursachen und Therapie* [Bulimia nervosa: Causes and therapy]. Springer, Berlin, Heidelberg.

[r66] Watson, D., Clark, L. A., & Tellegen, A. (1988). Development and validation of brief measures of positive and negative affect: The PANAS Scales. Journal of Personality and Social Psychology, 54(6), 1063–1070. 10.1037/0022-3514.54.6.1063 3397865

[r67] Zeeck, A., Herpertz-Dahlmann, B., Friederich, H. C., Brockmeyer, T., Resmark, G., Hagenah, U., Ehrlich, S., Cuntz, U., Zipfel, S., & Hartmann, A. (2018). Psychotherapeutic treatment for anorexia nervosa: A systematic review and network meta-analysis. Frontiers in Psychiatry, 9, 158. 10.3389/fpsyt.2018.00158 29765338 PMC5939188

[r68] Ziser, K., Mölbert, S. C., Stuber, F., Giel, K. E., Zipfel, S., & Junne, F. (2018). Effectiveness of body image directed interventions in patients with anorexia nervosa: A systematic review. International Journal of Eating Disorders, 51(10), 1121–1127. 10.1002/eat.22946 30189104

